# Surgery and Reason: The End of History and the Last Surgeon

**DOI:** 10.3390/jcm12175708

**Published:** 2023-09-01

**Authors:** Dimitrios E. Magouliotis, Thanos Athanasiou, Dimitrios Zacharoulis

**Affiliations:** 1 Unit of Quality Improvement, Department of Cardiothoracic Surgery, University of Thessaly, Biopolis, 41110 Larissa, Greece; 2 Department of Surgery and Cancer, Imperial College London, St Mary’s Hospital, London W2 1NY, UK; t.athanasiou@imperial.ac.uk; 3 Department of Surgery, University of Thessaly, Biopolis, 41110 Larissa, Greece; zacharoulis@uth.gr

Arguably, Georg Wilhelm Friedrich Hegel has been one of the most influential philosophers of the 19th century. In his Lectures on the Philosophy of World History [1], given at the University of Berlin between 1822 and 1830, he described world history not just as a sequence of random events but as rational progress toward a specific purpose. This purpose was identified as reaching the ultimate level of knowledge and freedom. In fact, in the introduction to these lectures, Hegel declared that there is reason in history and, vice versa, that world history is the progress of reason. However, reason also represents the moving force behind every progress and advance in the fields of medicine and surgery. Dating back to Hippocrates and the well-known phrase “Primum non nocere” or “First do no harm” reason in medicine and surgery mandates us not only to provide our best services to patients but, primarily, to provide them in a safe manner by creating and establishing a culture of safety. In other words, the reason that surgery passes through quality improvement (QI) in science.

QI and patient safety (PS) have become increasingly important in all surgical disciplines over the last two decades [2,3]. QI represents a continuous process whereby tools or methods are employed to promote measurable changes within a system which, in this case, is surgery [2,3]. QI interventions are at the core of this process, which is not a straight line but follows a spiral path of concentric circles dictated by reason (Figure 1). When a QI intervention is initiated, an established dogma is challenged. Through this clash of different theories, the practices associated with the best evidence-based outcomes prevail. A circle closes, and a new one opens with new clinical questions under examination. This process represents an analog to the progress of history proposed by Hegel, and we could admit that this is a process dictated by reason in surgery (Figure 1). 

Given the pivotal role of reason in surgery and QI, we should further stress this point. One of the primary vehicles of advancement in QI science is the Plan-Do-Study-Act (PDSA) scheme [4]. Passing through each one of the four steps of the PDSA cycle leads to the establishment of a new clinical practice pattern. The PDSA cycle represents the assessment of a clinical practice that is opposed or subsidiary to the previously established model. The outcomes of these two alternative practices are compared, and through this clash of ideas and theories, only the system providing the best outcomes for patients prevails. Probably a great example of this clash of ideas has been the use of multiple arterial grafts (MAG) instead of single arterial grafting (SAG) in coronary artery bypass grafting (CABG). Over the past few years, there has been growing evidence favoring the utilization of multiple arterial conduits in appropriate patients undergoing CABG [5,6,7]. However, the adoption of multiple arterial conduits utilization has been relatively slow [8]. In this context, QI interventions were designed and implemented by courageous surgical societies, such as the Michigan Society of Thoracic and Cardiovascular Surgeons Quality Collaborative (MSTCVS-QC) [9]. These initiatives paved the way for a significant increase in MAG adoption [10], thus enhancing outcomes and providing more data on long-term outcomes. Based on increasing evidence favoring the use of multiple arterial conduits in patients undergoing CABG [11], a “Hegelian” circle, based on the superiority of the MAG approach, is about to close, and a new one is about to open, which will examine different strategies in conduits harvesting, treatment protocols on the extent of target vessel stenosis for radial artery conduits, along with post-discharge treatment protocols.

The present Special Issue includes several articles that aim to answer important debates on different perioperative treatment pathways [12,13,14,15,16,17,18]. Two of them [14,15] validate risk-stratification tools, thus providing a necessary insight into preoperative planning and patient counseling while enhancing the shared decision-making process. In addition, Giardini et al. [12] compare two techniques in performing the supine-to-sitting postural change in patients with sternotomy, while Frisiras et al. [16] compare morbidity and mortality outcomes in elderly and nonelderly patients undergoing elective thoracic endovascular aortic repair (TEVAR). Such articles provide evidence that can enable the design and progression of different PDSA cycles, thus serving the unfolding of reason in surgical history.

Another core concept of the Hegelian dialectic is the provision of “world-historical individuals”, the so-called “great men” of history, such as Socrates or Julius Caesar. In this context, world-historical individuals are able to influence, guide the tides of history and drive it forward through their actions and initiatives, thus leading to higher levels of knowledge and freedom. In surgery, there are many examples of world-historical individuals. Dr. Denton Cooley and Dr. Michael DeBakey in cardiac surgery, along with Dr. David Sugarbaker in thoracic surgery (mesothelioma surgery), perhaps represent such figures. These great surgeons have opened new paths in surgery through their actions and initiatives. In the QI context, the existence of such world-historical individuals is even more important, given the complexity of the tasks they undertake. Dr. Richard Prager is a characteristic world-historical individual in the field of QI in cardiothoracic surgery. From the very beginning of his efforts to establish a QI program in the State of Michigan, Dr. Prager faced certain great challenges, such as a) gathering all cardiothoracic surgeons of the State around a common table to discuss their outcomes and designing QI initiatives, b) unblinding performance data at the independent-institution level, and c) partnering the MSTCVS-QC with a payer which, in that case, was the Blue Cross Blue Shield of Michigan (BCBSM): the state’s primary insurance payer [19]. Such disruptive individual actions are totally necessary for the progress of QI in surgery. In 1806, Hegel wrote a letter to his friend Friedrich Niethammer where he described Napoleon as “a world-soul [Weltseele] on horseback”, indicating Napoleon to be a world-historical individual that drove forward reason’s history. The well-known painting “Napoleon at the Saint-Bernard Pass” by Jacques-Louis David is the representation of Hegel’s idea of Napoleon. Perhaps we can declare that disruptive surgeons like Dr. DeBakey, Dr. Cooley, or Dr. Prager are real-life representations of “a world-soul with scrubs”. 

A final crucial question is whether there is an end to the progress of history, and what is that end? As previously commented, Hegel is using the word “history” as the unfolding of reason in the progress of the consciousness of freedom. This has led some intellectuals like Francis Fukuyama to declare that the goal of self-consciousness and human freedom has been achieved in recent times, and the world has reached “the end of history” [20]. In this context, what Hegel means by an end of history is that the goal of history has been achieved, and the world is now conscious of freedom instead of lacking any further developments. In the context of surgery, the end of history could be reached through the awareness and adoption of QI methodology by the surgical community in their practice as a veil of safety for patients. The “last surgeon”, the surgeon at the “end of history”, would implement these principles in his practice and actively take part in QI initiatives. Perhaps, we are not far from such an end to history, and possibly many among us, there tends to be a resemblance to the “last surgeon”. Nonetheless, the prevalence of such a heroic surgical idealism and culture in our time is totally necessary in order to protect and promote the best interests of patients, surgeons, and society as a whole.

## Figures and Tables

**Figure 1:**
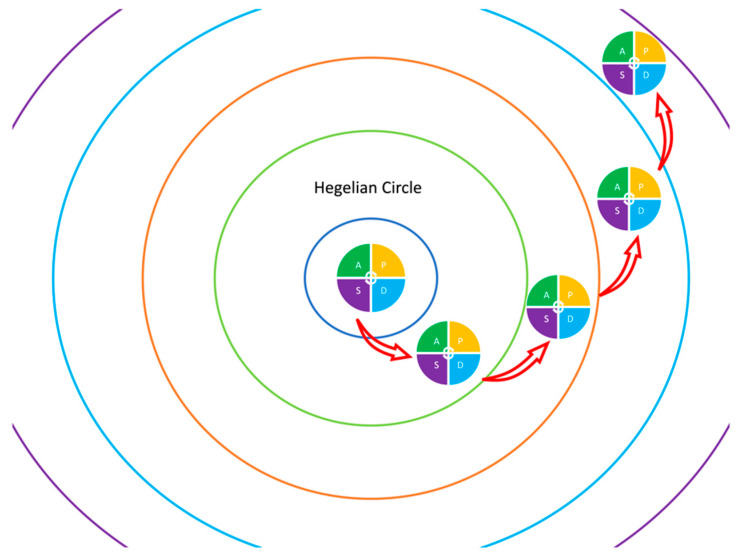
Representation of the merge between the Hegelian concentric circles of reason in history and the Plan-Do-Study-Act (PDSA) cycles of Quality Improvement in Surgery. This merge demonstrates the historical progress of reason in surgery.
